# A murine model of hypertensive heart disease in older women

**DOI:** 10.7717/peerj.17434

**Published:** 2024-05-23

**Authors:** Audrey Morin-Grandmont, Elisabeth Walsh-Wilkinson, Sara-Ève Thibodeau, Dominique K. Boudreau, Marie Arsenault, Yohan Bossé, Jacques Couet

**Affiliations:** Université Laval, Centre de Recherche, Institut Universitaire de Cardiologie et de Pneumologie de Québec, Québec, QC, Canada

**Keywords:** Hypertension, Aging, Heart failure, Woman, Mouse, Cardiac hypertrophy, Menopause, Ovariectomy, RNA sequencing

## Abstract

We propose a new mouse (C57Bl6/J) model combining several features of heart failure with preserved ejection fraction encountered in older women, including hypertension from Angiotensin II infusion (AngII), menopause, and advanced age. To mimic menopause, we delayed ovariectomy (Ovx) at 12 months of age. We also studied the effects of AngII infusion for 28 days in younger animals and the impact of losing gonadal steroids earlier in life. We observed that AngII effects on heart morphology were different in younger and adult mice (3- and 12-month-old; 20 and 19% increase in heart weight. *P* < 0.01 for both) than in older animals (24-month-old; 6%; not significant). Ovariectomy at 12 months restored the hypertrophic response to AngII in elderly females (23%, *p* = 0.0001). We performed a bulk RNA sequencing study of the left ventricle (LV) and left atrial gene expression in elderly animals, controls, and Ovx. AngII modulated (|Log_2_ fold change| ≥ 1) the LV expression of 170 genes in control females and 179 in Ovx ones, 64 being shared. In the left atrium, AngII modulated 235 genes in control females and 453 in Ovx, 140 shared. We observed many upregulated genes associated with the extracellular matrix regulation in both heart chambers. Many of these upregulated genes were shared between the ventricle and the atrium as well as in control and Ovx animals, namely for the most expressed *Ankrd1, Nppb, Col3a1, Col1a1, Ctgf Col8a1*, and *Cilp*. Several circadian clock LV genes were modulated differently by AngII between control and Ovx females (*Clock, Arntl, Per2, Cry2,* and* Ciart*). In conclusion, sex hormones, even in elderly female mice, modulate the heart’s hypertrophic response to AngII. Our study identifies potential new markers of hypertensive disease in aging female mice and possible disturbances of their cardiac circadian clock.

## Introduction

Heart failure with preserved ejection fraction (HFpEF) has emerged as an essential health problem with an increasing prevalence (Ponikowski et al., 2016). HFpEF, with the ongoing obesity and diabetes epidemics, now represents at least half of the HF cases. After the first hospitalization, the 5-year survival is less than 50%. Patients with HFpEF are usually older, many of them female and obese, and exhibit a lower prevalence of coronary artery disease than patients with heart failure with reduced ejection fraction (HFrEF) (Kitzman & Shah, 2016). Patients with HFpEF have higher morbidity, mortality, and rehospitalization than those with HFrEF and poorer quality of life (Shah et al., 2016b). The complex interaction between aging and comorbidities thus makes HFpEF a substantial burden on healthcare systems since evidence-based treatments are still to be developed (Savarese & Lund, 2017; Dunlay, Roger & Redfield, 2017; Virani et al., 2021).

Old age, hypertension, atrial fibrillation, and the female sex are classical factors for the development of HFpEF. This HFpEF phenotype is characterized by left ventricle (LV) concentric remodelling, left atrial enlargement, and high levels of natriuretic peptides (Cohen et al., 2020). A relatively novel cardio-metabolic phenotype where the proportion of male patients is more elevated, including obesity and type 2 diabetes mellitus, has become more prevalent in recent years (Ho et al., 2016; Shah et al., 2016a). Most HFpEF patients have a history of hypertension. Proper and adequate management of this condition was shown to reduce its incidence by up to 40% (Redfield & Borlaug, 2023; Sciarretta et al., 2011; Upadhya, Stacey & Kitzman, 2019). Other pro-inflammatory comorbidities such as obesity or diabetes mellitus (present in almost 70% of patients) may promote HFpEF through cardiac microvascular endothelial cell inflammation and increased oxidative stress. Extensive clinical trials have yet to investigate anti-inflammatory drugs. Clinical trials of agents treating impaired nitric oxide signalling have been negative (Shah et al., 2020). Thus, the role of inflammation and reduced nitric oxide bioavailability, although likely in the development of HFpEF, remains unclear.

Many factors contribute to the predisposition of older women to develop HFpEF (Beale et al., 2018). These include age-associated changes in cardiovascular physiology, arterial stiffness, preservation of LV mass and systolic function with reduced diastolic function and enhanced inflammatory responses in older women. The high prevalence of unmanaged hypertension, obesity, hypertension, diabetes, and other comorbidities further contribute to this risk, as mentioned above (van Ommen et al., 2022). Given the limited current therapeutic options for HFpEF in either sex, heightened efforts to develop new prevention and treatment strategies are needed.

The availability of suitable HFpEF animal models reflecting these different phenotypes is essential to explore new pathophysiological mechanisms and to test new therapeutic approaches. In the mouse, HFpEF models triggered by a single factor (hypertension, obesity/diabetes, or aging) are currently replaced by more complex models combining more than one factor (Withaar et al., 2021a). Schiattarella et al. (2019) reported inducing HFpEF in young male mice by combining metabolic stress (high-fat diet (HFD)) and hemodynamic stress (hypertension induced using a nitric oxide synthase inhibitor). Withaar et al. (2021b) proposed a model combining the feeding of an HFD and angiotensin II (AngII) infusion in aged female mice. Interestingly, the model developed in young mice only worked in males, whereas in old mice, it worked only in females. Males, unlike females, displayed features of heart failure with reduced ejection fraction when subjected to HFD and AngII (Tong et al., 2019). Matsiukevich et al. (2023) described a new mouse model of HFpEF by targeting the *α*1 adrenergic receptor with an agonist, phenylephrine, and AngII in young animals. This model displayed many features associated with HFpEF. The study used male and female animals but did not emphasize sex-based differences. We recently proposed our HFpEF mouse model, which combined AngII and HFD for four weeks, and it was amenable in both young and older animals, either male or female (Aidara et al., 2024).

These studies suggest that consideration for age, biological sex, and/or sex hormones are all critical factors worth considering in the development of HFpEF mouse models. In addition, since HFpEF is more prevalent in the aging population and more than twice as often in postmenopausal women, the absence of ovarian hormones is also likely to affect the development of HFpEF (Sabbatini & Kararigas, 2020; Maslov et al., 2019). Ovariectomy is frequently used as a surrogate for menopause in preclinical models (Bhuiyan & Fukunaga, 2010). To our knowledge, no study has studied the effects of the loss of sex steroids in mice, if delayed later in life, on heart morphology and function in aging females and the impact of hypertensive stress in those animals.

We investigated the effects of a continuous infusion of angiotensin II (AngII) in older female mice (ovariectomized (Ovx) or not). We show that the loss of gonadal steroids influences the heart’s response to hypertensive stress. Using bulk left ventricle RNA sequencing; we also identified several new genes associated with cardiac aging and the reaction to hypertension in older female mice.

## Materials and Methods

### Animals

C57BL6/J female mice were purchased from Jackson Laboratory (Bar Harbor, ME, USA) or the aging colony of the Quebec Aging Research Network (RQRV) and were kept for up to 24 months of age. Mice were housed on a 12 h light-12 h dark cycle with free access to chow and water. The protocol was approved by the Université Laval’s animal protection committee and followed the recommendations of the Canadian Council on Laboratory Animal Care (#2019-075 and #2020-603). This study was conducted following the ARRIVE guidelines.

### Experimental design

**Figure 1:**
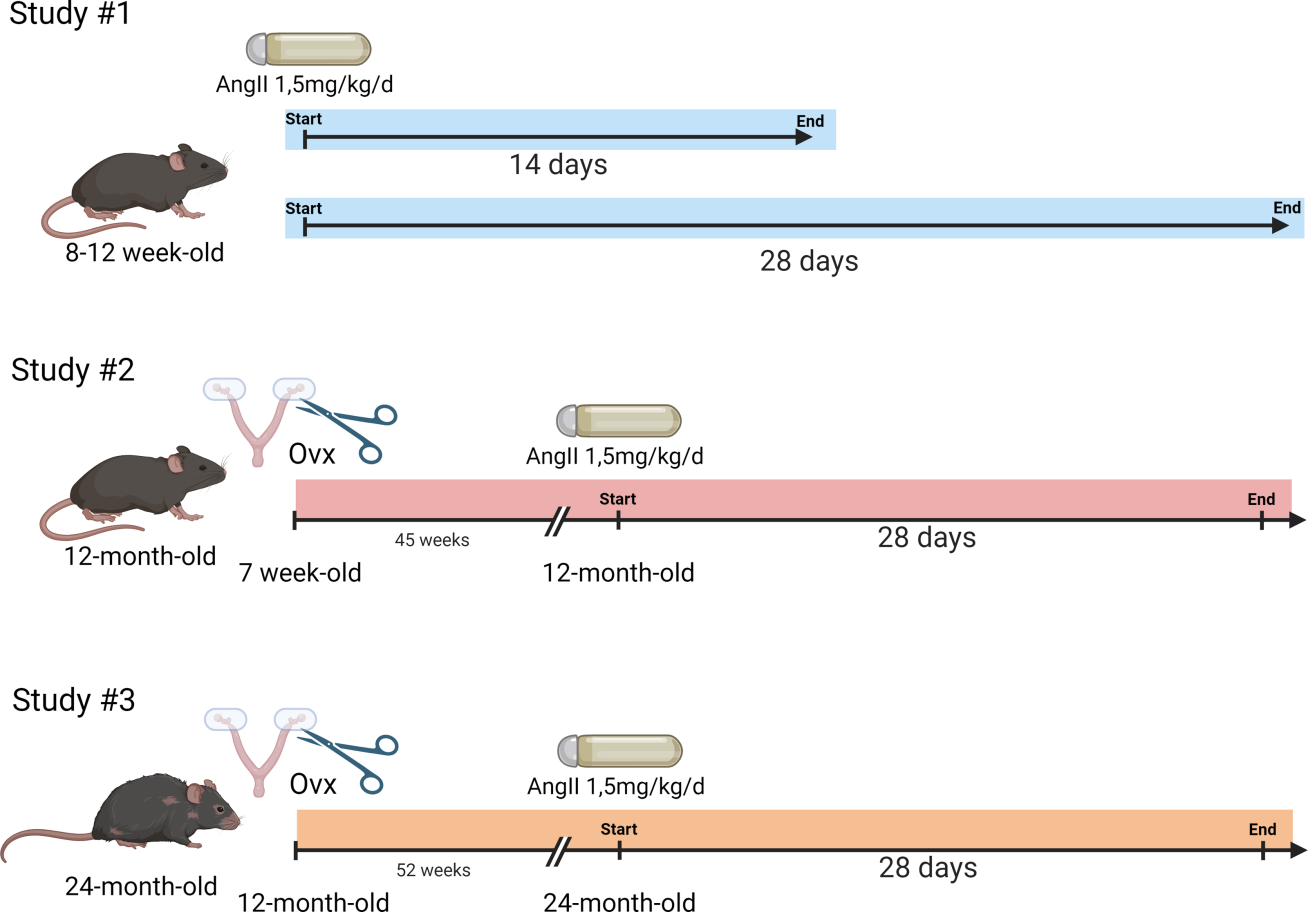
Schematic representation of the design of the animal study. Study #1. Eight-week-old mice were treated or not with AngII for 14 or 28 days (*n* = 8 per group). Study #2. Seven-week-old mice were ovariectomized or not. Then, at 12 months, AngII treatment was performed for 28 days (*n* = 8/AngII group and *n* = 8/control group). Study #3. Twelve-month-old mice were ovariectomized or not. At 24 months, AngII treatment was performed for 28 days (*n* = 7–8/ group). Created with BioRender (https://www.biorender.com/).

Mice were randomly distributed in the various aging groups. The general design of the study is illustrated in Fig. 1.

Study #1: Three-month-old mice. Twenty-four 7-week-old mice were equally and randomly distributed between the following experimental groups (*n* = 8): untreated or treated with AngII for 14 or 28 days). The mice had an echocardiography exam the day before euthanasia.

Study #2: Twelve-month-old mice. Thirty-two 7-week-old mice were distributed between four groups. Sixteen mice were ovariectomized at seven weeks. Eight ovariectomized mice and eight controls were treated with AngII for 28 days at 12 months. The day before euthanasia, all mice had an echocardiography exam.

Study #3: Twenty-four-month-old mice. Thirty-two 12-month-old mice were distributed between four groups (*n* = 8): Ovx at 12 months or not. Half of these mice were treated with AngII starting at 23 months and lasting 28 days. Blood pressure was taken one week before the end of the protocol, and all mice had an echocardiography exam the day before euthanasia.

All mice treated with AngII underwent the same procedure: An ALZET osmotic minipump (Model 2004) delivering a continuous infusion of AngII (1, 5 mg/kg/day) was implanted subcutaneously on their backs under isoflurane-induced anesthesia (Walsh-Wilkinson et al., 2022). All mice were weighed weekly.

Animal behaviour was monitored daily by experienced technicians for health and behaviour during the protocol. We immediately euthanized mice displaying signs associated with poor prognosis of quality of life or specific signs of severe suffering or distress. Among those signs, significant loss or gain of weight, palpable tumours, grooming, and changes in behaviour were recorded. One animal (24 months + AngII) was sacrificed before the termination of the protocol. All older mice over 12 months of age had available in their cage a running saucer (Innowheel™; Innovive, Billerica, MA, USA) as an environmental enrichment to avoid unwanted behaviours such as barbering.

Euthanasia was performed by total exsanguination of isoflurane-anesthetized animals followed by cervical dislocation between Zeitgeber (Zt) hours 2 and 6. Hearts were quickly removed, rinsed in phosphate-buffered saline, and weighed. The left atrium was then dissected and weighed. Lungs were also weighed.

### Blood pressure

Blood pressure was measured using the non-invasive CODA tail-cuff system (Kent Scientific, Torrington, CT, USA). Briefly, each mouse was placed in a clear mouse holder and onto a warming platform for the duration of the experiment. The cuffs were placed around each mouse’s tail, and the experiment began when the animals were calm. Mice were acclimated to the holder for 15 min thrice on consecutive days before the final measurements were taken. Twenty measures were taken, and the mean blood pressure for each mouse was calculated. The lights of the procedure room were dimmed to reduce stress, and body temperature was monitored. Blood pressure was measured three weeks after the beginning of AngII treatment.

### Echocardiography

The same investigator, blinded for mouse identification, acquired Echo images on a Vevo 3100 imaging system (VisualSonics, FujiFilm, Toronto, Canada). Transthoracic echocardiography was performed using a 40 MHz image transducer (MX550S) as previously described (Walsh-Wilkinson et al., 2022; Walsh-Wilkinson, Arsenault & Couet, 2021).

### Histological analysis

Tissue preparation. OCT-embedded frozen cardiac tissue sections (long axis) were cut 10 µm thick and fixed on a microscope slide. LV sections were fixed using Bouin’s solution overnight, and the nuclei were stained using Wiegert’s hematoxylin for 10 min. Fibrosis was then stained using Picrosirius Red dye for 1 h. Images of each section of the LV (interventricular septal wall (IVS), apex, posterior or free wall, and LVPW) were acquired using a wide-field microscope (Zeiss). Each of these sections was then analyzed using GIMP (GNU Image manipulation program; https://www.gimp.org/). Both microscopy and image analysis were performed by investigators who were blinded for mouse identification.

LV fibrosis assessment. Three sections of the LV (septum, apex, and free wall) were independently analyzed, and the mean percentage of interstitial fibrosis of the three sections was determined. Briefly, LV fibrosis of each section was measured by calculating the ratio of stained (red) pixels to the total number of pixels representing cardiomyocytes and fibrosis. The number of pixels corresponding to white sections (holes in the tissue) was measured and deducted from the total number of pixels of the image to get the total number of pixels representing the myocardium. Measures were acquired using the “by colour select” tool in the software.

Cardiomyocyte cross-sectional area (CSA). When acquiring the images, an LV image on which cardiomyocytes were easily visible and in the “en face” position was explicitly taken for cardiomyocyte CSA measurements. A total of five cardiomyocytes were measured. The mean was calculated for each sample. Cardiomyocyte CSA was measured in pixels using the “free select” tool in the software. The area expressed in pixels was then converted to µm^2^ by multiplying the number of pixels for one cardiomyocyte by the size in µm^2^ of a single pixel. The microscope software automatically calculates a single pixel’s width and length in µm.

### RNA preparation for mRNA sequencing

Total RNA was extracted from cardiac tissue samples using TRI Reagent (Sigma, Mississauga, Ontario, Canada). RNA from the LV was extracted for all 24-month-old experimental groups, and RNA was also extracted from the left atrium (LA) for all 24-month-old females. Samples were then treated with DNase I (RapidOut DNA removal kit; Thermo Fisher Scientific, Waltham, MA, USA), and RNA integrity was analyzed with the Agilent 2100 Bioanalyzer and RNA 6000 Nano kit.

### Bulk mRNA-sequencing

For LV samples, four pools of total RNA from two mice for each experimental group were used to create libraries using Stranded mRNA Prep and Ligation kit (Illumina) following the manufacturer’s recommendations. Libraries from LA RNA were made using four samples for each experimental group. Libraries were also analyzed using the Agilent 2100 Bioanalyzer and DNA 1000 kit. All samples were then quantified using the KAPA Library Quantification Kit (Kapa Biosystems, Wilmington, MA, USA). The average size of libraries was 335 base pairs. Indexed libraries were pooled and sequenced on the Illumina NextSeq 2000 (paired-end 150 bp) using sequencing-by-synthesis chemistry v4 according to the manufacturer’s protocols. We averaged 8.0 × 10^7^ (3.7 to 15.3 × 10^7^) paired-end reads, with an average of >95% of the reads achieving a quality score equal to or greater than Q30 for the LV samples. For the left atrial libraries, the average reads were 6.1 ×10^7^ (5.0 to 7.6 × 10^7^), with an average of >95% of the reads achieving a quality score equal to or greater than Q30 for the LA samples. We performed a power analysis calculation and found that the statistical power of this experimental design, calculated in RNASeqPower, required an n of 2.48 (https://rodrigo-arcoverde.shinyapps.io/rnaseq_power_calc/). The factors used were a 6 × 10^7^ value of sequencing death in base pairs, a coefficient of variation of 0.2, an effect of 2, an alpha of 0.05, and a power of 90%. We used the Illumina BaseSpace RNA-Seq Alignment (STAR) RNA-Seq Differential Expression (DESeq2) software for sequence alignments and identifying differentially expressed genes (DEG) using the Mus musculus UCSC mm10 reference genome. The DEGs were determined by adjusting the *p*-value for multiple tests using Benjamini–Hochberg correction with false discovery rate (FDR) ≤ 0.05 and Log2 fold change (Log2FC), —Log2 FC—≥ 1.0. Gene set enrichment analysis was performed using the Panther GO Enrichment Analysis (https://geneontology.org/) (Thomas et al., 2022; Ashburner et al., 2000; The Gene Ontology Consortium, 2023). A differentially expressed genes list (FDR <0.05 and —Log2FC—≥ 1.0) was used to perform this analysis. Significant GO terms were determined using Fischer’s Exact test using Benjamini–Hochberg correction with FDR. GEO accession numbers: GSE240171, GSE250052, and GSE250053.

### Statistical analysis

All data are expressed as mean ± standard error of the mean (SEM). Intergroup comparisons were conducted using the Student’s *T*-test using GraphPad Prism 9.5 (GraphPad Software Inc., La Jolla, CA, USA). Outliers were detected using the ROUT test (GraphPad Prism). Comparisons of more than two groups were analyzed using one-way or two-way ANOVA and Holm-Sidak post-test. *P* < 0.05 was considered statistically significant. Raw data are provided as Supplemental Data.

## Results

### A 28-day-long Angiotensin II infusion does not induce cardiac hypertrophy in 24-month-old non-Ovx female mice

Three-month-old, twelve-month-old, and twenty-three-month-old female mice (Ovx at 12 months or not) received for 28 days a continuous infusion of Angiotensin II (1.5 mg/kg/day) administered *via* a micro-osmotic pump implanted subcutaneously in the neck. Younger mice also received a shorter AngII infusion of 14 days. Ovariectomized (Ovx) mice were also studied. Twelve-month-old mice had been Ovx at the age of 7 weeks, whereas those aged 23 months were Ovx at the age of 12 months. We pursued two goals for the study of Ovx mice. The first is to investigate the effects of AngII long after the loss of gonadal steroids, 10 and 11 months after the Ovx, respectively. The second was to study the differences between Ovx at a young age during adolescence or young adulthood in females or later in life in adult mice where reproductive capacity has already significantly declined to simulate menopause better.

**Figure 2:**
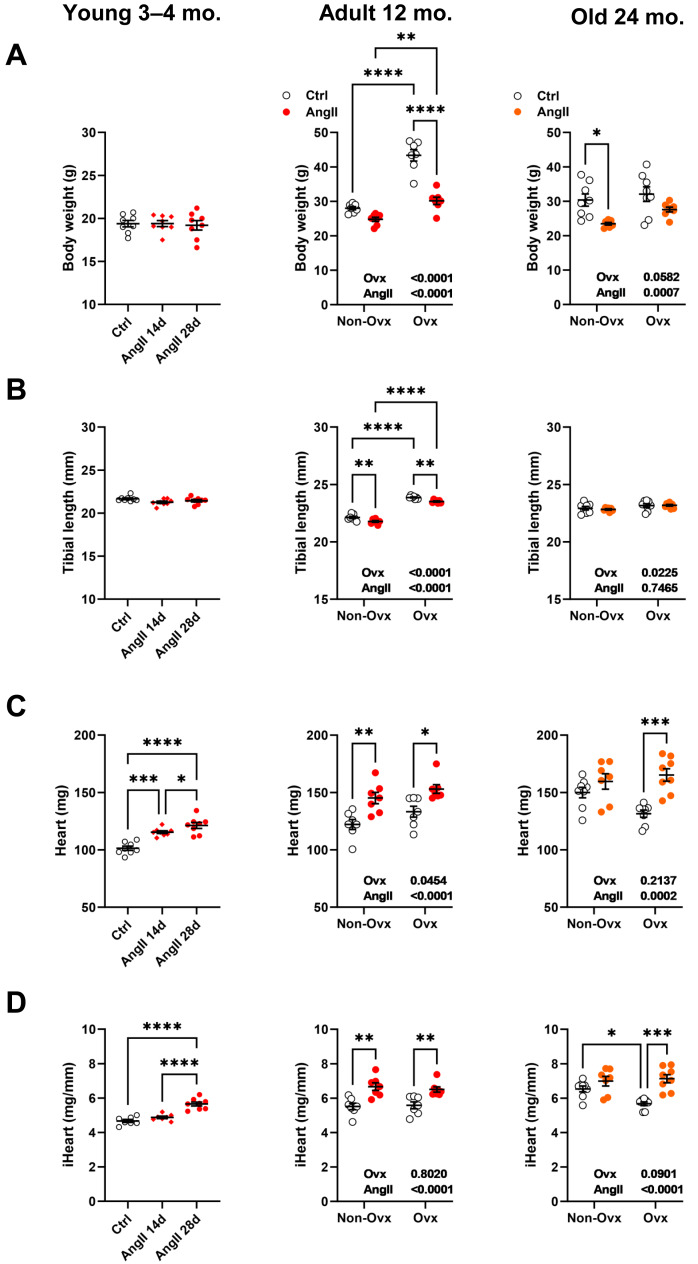
Effects of an Angiotensin II (AngII) on the heart. Graphs of young (3–4-month-old), adult (12 months), and elderly (24 months) are represented side by side for each panel. Ovariectomized mice (Ovx) and days (d). (A) Body weight, (B) tibial length, (C) heart weight, (D) indexed heart weight for tibial length (iHeart). Data are represented as the mean +SEM (*n* = 7–8 mice/group). One-way (young) or Two-way (adult and old) ANOVA followed by the Holm-Sidak pos *t*-test. Significant two-way ANOVAs are indicated in the graphs for adult and old mice. *: *p* < 0.05, **: *p* < 0.01, ***: *p* < 0.001 and ****: *p* < 0.0001 between indicated groups.

As illustrated in Fig. 2A, Ovx increased body weight in 12-month-old mice compared to control animals. Increased body weight after Ovx at 12 months was not observed in elderly mice. AngII markedly decreased body weight in 12-month-old Ovx animals and 24-month-old control (Ctrl) mice. In younger mice, no effects of AngII on body weight were observed. We used tibial length as a marker of overall body growth (Fig. 2B). Again, in younger animals, AngII had no effects. However, AngII significantly reduced tibial length in 13-month-old females. Ovariectomy of young mice resulted in significantly longer tibial length in adult mice, whereas Ovx, later in life, had a more minor effect.

Angiotensin II infusion resulted, as expected, in cardiac hypertrophy (Figs. 2C and 2D). This was true for young mice treated for either 14 or 28 days and older mice except for 24-month-old control mice, where AngII did not result in additional cardiac hypertrophy over the one resulting from aging. Interestingly, if AngII caused similar levels of cardiac hypertrophy in 13-month-old mice, in older animals, Ovx stopped cardiac hypertrophy related to aging during the second year of life but kept the heart’s capacity to respond to AngII.

### Angiotensin II infusion increases LV wall thickness in 24-month-old Ovx female mice but not in controls

**Figure 3:**
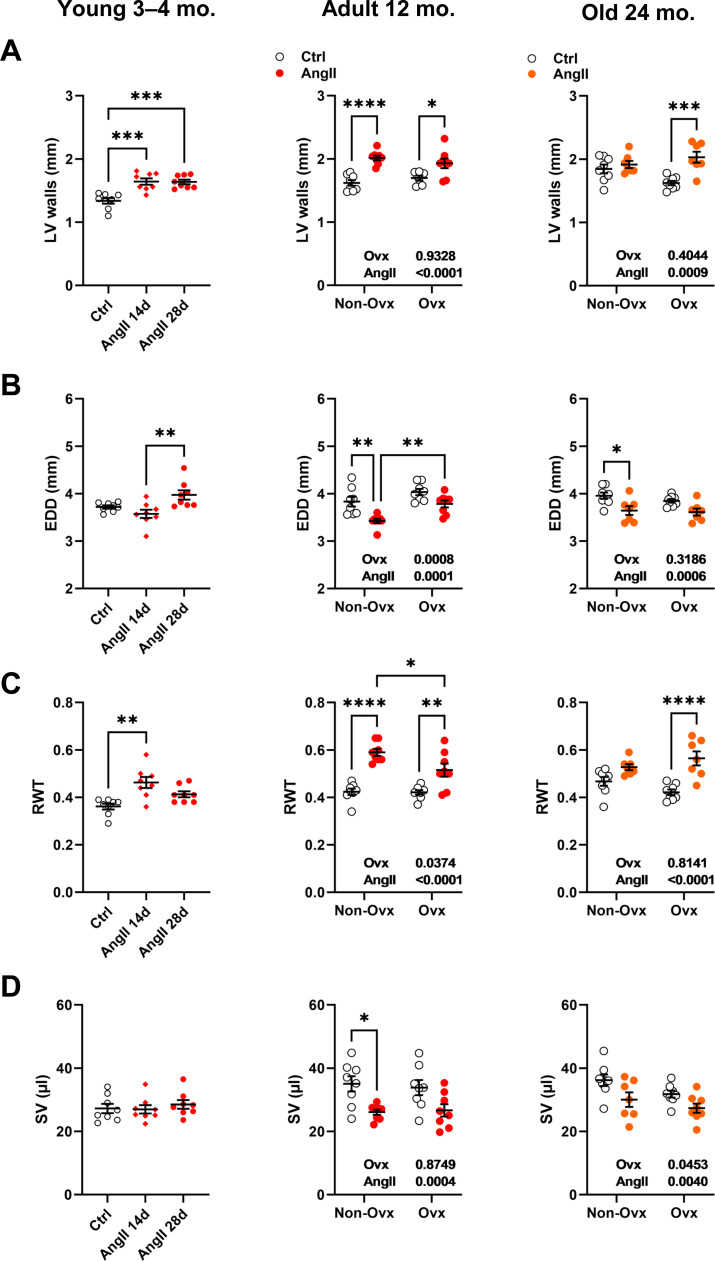
Effects of an Angiotensin II (AngII) on various left ventricle echocardiography measurements. Graphs of young (3-4-month-old), adult (12 months), and elderly (24 months) are represented side by side for each panel. Ovariectomized mice (Ovx) and days (d). (A) LV walls; posterior LV wall thickness + interventricular septal wall thickness, (B) EDD; end-diastolic LV diameter, (C) RWT; relative wall thickness and D: SV; LV stroke volume Data are represented as the mean +SEM (*n* = 7–8 mice/group). One-way (young) or Two-way (adult and old) ANOVA followed by Holm-Sidak pos *t*-test. Significant two-way ANOVAs are indicated in the graphs for adult and old mice. *: *p* < 0.05, **: *p* < 0.01, ***: *p* < 0.001 and ****: *p* < 0.0001 between indicated groups.

LV wall thickness remained unchanged after AngII in elderly control females but increased in Ovx animals (Fig. 3A). AngII caused LV walls to thicken in all younger animals. The effects of AngII and/or Ovx on end-diastolic LV diameter (EDD) were different in young mice, where they tended to increase but were reduced in older animals (Fig. 3B). In 12-month-old and elderly mice, EDD was smaller in control animals than in Ovx mice. This resulted in a slight and non-significant increase in RWT in control females and a marked one in the Ovx group in elderly mice (Fig. 3C). AngII increased relative wall thickness in 12-month-old mice but more so in controls. Left ventricle stroke volume (SV) remained unchanged in younger animals receiving AngII but was reduced in older animals (Fig. 3D). Detailed echo data are listed in Tables S1 to S3. Representative M-mode echo views of young and aged mice LV and the effects of AngII are illustrated in Fig. S1.

### Angiotensin II infusion worsens diastolic function in 24-month-old mice

**Figure 4:**
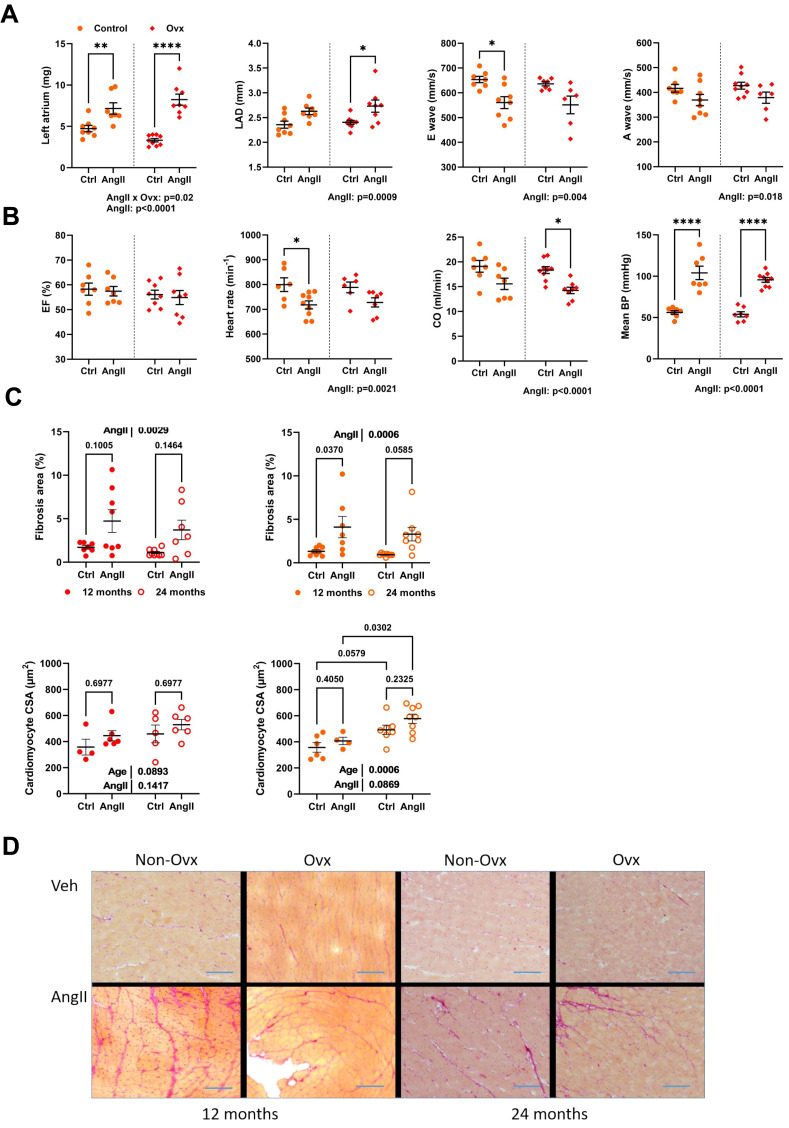
Angiotensin II infusion in elderly mice is accompanied with LV diastolic abnormalities and increased interstitial myocardial fibrosis. (A) From left to right. Left atrial weight at euthanasia, left atrial diameter by echocardiography, as well as E and A waves through the mitral valve. (B) Ejection fraction (EF), Heart rate in conscious mice, Cardiac output (CO), and mean blood pressure (BP). (C) Up. AngII infusion increased interstitial fibrosis area (%) in 12- and 24-month-old control (left; red) and Ovx (right; orange) mice. Down. Cardiomyocyte cross-sectional area in the same animals. (D) Representative images of picrosirius red-stained LV sections. Bar: 100 µm. Data are represented as mean +SEM (*n* = 7-8 mice/group). Two-way ANOVA followed by Holm-Sidak post-test. *: *p* < 0.05, **: *p* < 0.01, ***: *p* < 0.001 and ****: *p* < 0.0001 between indicated groups.

AngII caused left atrial hypertrophy for both control and Ovx elderly females, suggesting diastolic dysfunction (Fig. 4A). Left atrial diameter, E, and A waves were also modulated by AngII (Fig. 4A). AngII did not change ejection fraction, although cardiac output was decreased. It reduced heart rate in conscious mice, and AngII increased blood pressure as expected (Fig. 4B).

Interstitial LV fibrosis was increased in AngII females (Figs. 4C–4D). LV fibrosis was not more abundant in older mice than those aged 12 months. AngII increased the cardiomyocyte cross-sectional area. Results were similar in Ovx mice (Fig. 4D).

### Angiotensin II strongly induces genes related to the extracellular matrix in the left ventricle and the left atrium in elderly mice, ovariectomized or not

**Figure 5:**
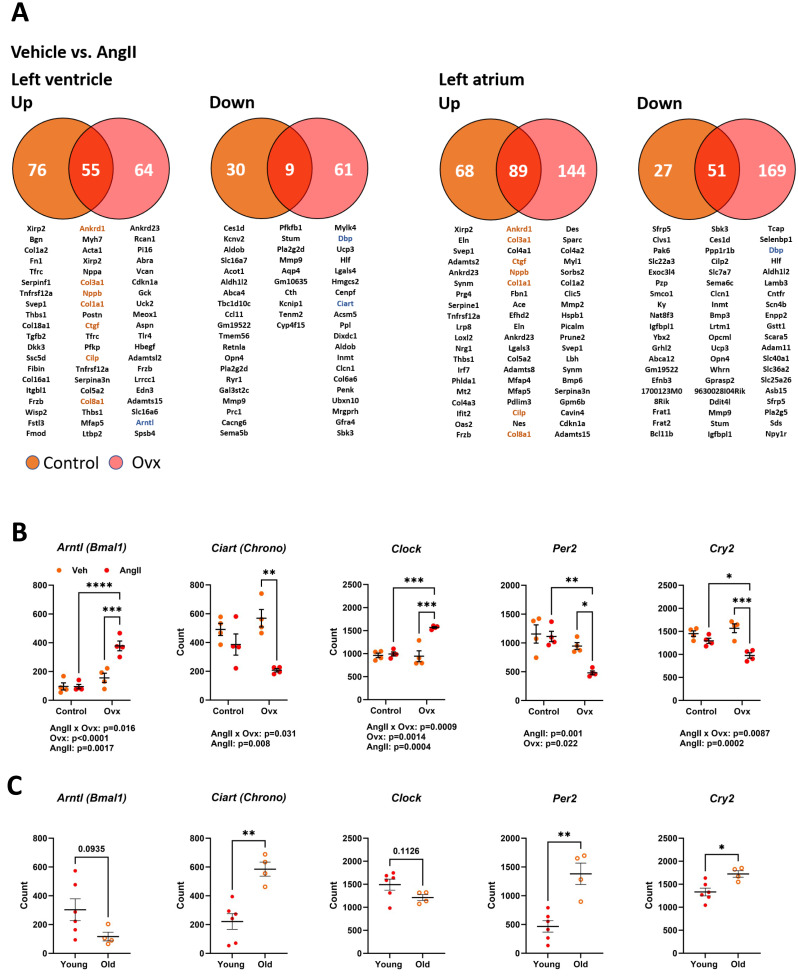
Transcriptomic changes in the left ventricle induced by Angiotensin II (AngII) in old female mice. Disturbances in circadian clock genes. (A) Differentially expressed genes between control (orange) and Ovx (red) mice treated with AngII for 28 days. Venn diagrams of up (left) or down-regulated (right) genes in the left ventricle and left atrium. Below, circles and the intersection of the diagrams are listed up to twenty genes with the highest expression levels for each comparison. In orange, common genes between the left ventricle and left atrium. (B) Gene expression of five core circadian clock genes in the left ventricle of old female mice treated or not with AngII. *Arntl*: Basic Helix-Loop-Helix ARNT Like 1, *Ciart*: Circadian Associated Repressor of Transcription, *Clock*: Clock Circadian Regulator, *Per2*: Period Circadian Regulator 2 and *Cry2*: Cryptochrome Circadian Regulator 2. (C) Core circadian clock gene expression in young and old male and female mice. Results are expressed as the mean ±SEM (*n* = 4). Statistical analysis uses two-way ANOVA followed by the Holm-Sidak pos *t*-test (B) or Student *T*-test (C). *p* values are indicated when under 0.05 below the graphs. *: *p* < 0.05, **: *p* < 0.01, ***: *p* < 0.001 and ****: *p* < 0.0001 between indicated groups.

As illustrated in Fig. 5A, 170 genes in elderly control mice and 189 in elderly Ovx animals receiving AngII for four weeks saw their expression doubled or halved in the left ventricle posterior wall. Over 65% of these modulated genes were upregulated. In the left atrium, only one gene, *Ltbp2* (+2.3-fold-change over control), was found to be differentially expressed between control and Ovx mice. A total of 196 genes in controls and 303 in Ovx receiving AngII for four weeks were modulated in the left atrium. Around 75% of these modulated genes were upregulated. As listed in Tables S4 and S5, AngII produced a DEG enrichment for biological processes and cellular components related to remodelling the extracellular matrix and myocardial fibrosis. As described in Fig. 5A, *Ankrd1, Col1a1, Col3a1, Col8a1 and Ctgf,* genes implicated in controlling the extracellular matrix or the production of its component were upregulated in both the atrium and the ventricle. This correlates with increased interstitial myocardial fibrosis, as shown in Figs. 4C–4D.

Several genes associated with the circadian clock were expressed differently after AngII infusion between Ovx and non-Ovx mice. As illustrated in Fig. 5B, the four core circadian clock genes, *Clock, Arntl, Per2, and Cry2*, were modulated in the LV of Ovx mice after AngII but not in control mice. We also looked for the influence of age on these circadian clock genes in the LV. Except for *Arntl* and *Clock*, they were up- or downregulated during aging (Fig. 5C). As illustrated in Fig. S2, several other circadian clock regulators or output genes, such as *Nr1d1 (Rev-Erb α*), *Dbp*, or *Npas2*, were also modulated by aging. The first two showed different expression levels in Ovx mice, and AngII modulated LV Ovx mice levels of *D* BP and *Npas2*. The expression of left atrial core circadian clock genes was much lower than in the LV. These levels were below the mean count threshold we had fixed to categorize genes as low expressers (below 50). Loss of estrogens was observed to modulate three of them, namely *Clock, Arntl*, and *Per2* (Fig. S3).

## Discussion

In this study, we investigated the relative contribution of age and loss of gonadal steroids at different times of life on heart morphology and function after hypertensive stress. Our objective was the development of a new murine model of hypertensive disease in older women by combining loss of sex steroids, age, and hypertension. For this, we used Ovx (at 12 months) mice receiving a 4-week Ang II infusion at 23 months. These mice had preserved ejection fraction and showed significant cardiac hypertrophy, LV concentric remodelling, myocardial fibrosis, and diastolic dysfunction (enlarged left atrium and changes in echo diastolic parameters). Angiotensin II infusion decreased body weight, most likely by reducing adipose tissue mass and skeletal wasting (Sugiyama et al., 2012). Interestingly, the body weights of Old Ovx mice treated with AngII were less reduced than those of non-Ovx mice, although, at the start of treatment, body weights were similar between the two groups.

Ovariectomy of rodents has often been used as a surrogate for menopause in preclinical models (Joll 2nd et al., 2022). Perhaps surprisingly, the effects of Ovx later in life have received very little attention in the past (Kyryk et al., 2014; Tomicek et al., 2013; Kyryk et al., 2023). Here, we observed that loss of ovarian steroids in 12-month-old females led to a stoppage of ongoing age-related cardiac hypertrophy and less left ventricle concentric remodelling. Twelve-month-old mice are believed to correspond to humans in their forties (Dutta & Sengupta, 2016).

Ovariectomy in 12-month-old females did not result in increased body weight gain compared to age-matched control females as for younger Ovx animals, which became obese. Another factor had to be considered here. We did not impose a sedentary lifestyle on aging mice to avoid abnormal behaviours that could result in the loss of animals for reasons unrelated to cardiac issues. Ovx-related obesity observed in young mice may have been reduced by voluntary exercise in older females. We did not monitor the running activity of our mice, so we do not know if Ovx mice had a different running behaviour than control females. We removed the running saucers during AngII treatment so that the cardiac remodelling would not be influenced by exercise. Ovx mice displayed a solid response to AngII, resulting in marked LV wall thickening. Concentric LV remodelling exceeded the one observed in control mice. This is an interesting feature of our model since changes observed in the left ventricle wall thickness are known to be accelerated in women, especially in hypertensive conditions and/or diabetes (Cheng et al., 2010). This concentric remodelling with aging in women is believed to favour the development of diastolic dysfunction and HFpEF (Cheng et al., 2010; Merz & Cheng, 2016; Gori et al., 2014). In addition, we showed that hypertensive stress was associated with the activation with the upregulation of genes related to the extracellular structure organization (17 to 28 genes, depending on the comparison), as described in Tables S4 and S5. The observed increase in interstitial myocardial fibrosis can explain the worsening of diastolic function.

Thus, we observed that cardiac hypertrophy and remodelling are ongoing in female mice influenced by age and sex steroid hormones. The result has similarities to one observed in older women, *i.e.,* CH, LV concentric remodelling, increased myocardial fibrosis, and cardiomyocytes CSA. Ovx resulted in a somewhat less severe LV concentric phenotype, but the capacity of the myocardium to remodel after a hypertensive insult was preserved.

One important addition that our model will probably need in the future is the inclusion of a metabolic stressor. Previous publications of HFpEF murine models (Withaar et al., 2021a; Schiattarella et al., 2019; Withaar et al., 2021b; Tong et al., 2019; Matsiukevich et al., 2023; Aidara et al., 2024) added a high-fat diet to hypertensive stress. We recently observed in young female mice that the high-fat diet alone was sufficient to cause significant levels of CH, comparable to AngII. This was not the case for male mice (Sabbatini & Kararigas, 2020). It will be engaging in the future to study the effects of a high-fat diet in Ovx old mice receiving or not AngII.

Unfortunately, our RNA-Seq data did not reveal many genes differentially expressed between control and Ovx females. This may be linked to the fact that by analyzing the LV transcriptome one year after Ovx, we have missed many earlier gene expression adaptations after removing gonadal steroids.

Our animals were not tested for diminished exercise capacity, and lung weight was not increased, so it may be premature to define our mouse model as an HFpEF model. Several HFpEF features were present, however. Compared to controls, cardiac hypertrophy, LV wall thickening, and LA hypertrophy were increased in Ovx-old females receiving AngII. Withaar et al. (2021b), in their HFpEF murine triple-hit model in aging females, added a high-fat diet for several months before AngII. Deng et al. (2021) used another approach by introducing a high-fat diet at a relatively young age (3 months) and for an extended period of 12 months, then administering the mineralocorticoid, deoxycorticosterone pivalate, for an additional month. In this study, sex-based differences were not studied. In these two HFpEF models, a combination of age, hypertension, and a high-fat diet combined various risk factors to mimic the situation in human patients. However, the effects of sex hormones were not assessed. Menopause, as understood by women, is not present in most mammals. In mice, ovaries do not fail with age as in women, but the uterine capacity to sustain pregnancies declines over time (Finn, 2001). The outcome is similar for the reproduction capacity. However, the impact of ovarian steroids continues in mice later in life, and our results suggest that their contribution must be considered.

Angiotensin II infusion had different effects depending on the age of female mice. In young mice, a gain of heart mass was accompanied by concentric LV remodelling. In 12-month-old mice, this mass gain was less pronounced, but concentric LV remodelling was increased. At 24 months, AngII did not cause additional cardiac hypertrophy or wall thickening. Instead, the LV chamber decreased in volume. This suggests that aging alone in female mice will lead to a near-maximal gain of cardiac mass, limiting the LV to gain further upon an insult such as hypertension. Ovariectomy later in life slowed cardiac hypertrophy related to aging but allowed a hypertrophic response to AngII with a small LV chamber and thickened walls. Blood pressure measured in conscious aged female mice was low, which can be related to a more keratinized tail and a dampened signal recorded by the plethysmograph. Still, AngII significantly raised BP. The heart of Ovx mice responded strongly to this pro-hypertrophic agent. It is unlikely that in control females, pressure overload from higher BP was insufficient to result in a hypertrophic, suggesting that the myocardium of old aging Ovx females could be more sensitive to a hemodynamic insult. As mentioned above, it is also possible that the hearts of control-aged females already had reached near-maximal hypertrophy and concentric remodelling levels.

In our search of genes differently modulated between control and old Ovx females, we observed that the core circadian clock gene *Arntl* (*Bmal1*) expression was reduced with age and stimulated after AngII but only in Ovx mice. This was true for several other circadian clock genes. Our study was not designed to study the circadian clock genes, and the sacrifices of the animals were not done at the same time of the day. Euthanasia was, in general, conducted over 4 h during the day (Zt2- Zt6). Our RNA-seq data are from an RNA pool from two animals, which could reduce the extent of this limitation. A recent study has shown that the heart has 255 genes with a circadian pattern of expression, which had their levels modulated by aging in male mice. Unfortunately, females were not studied. This number of genes was relatively low compared to other tissues studied (hypothalamus, lung, kidney muscle) (Wolff et al., 2023). The authors suggested that the vital role of the heart may not permit important variations with aging.

It was unsuspected that AngII infusion in old Ovx mice would tend to normalize the expression of clock genes towards levels measured in young females instead of the opposite. We recently observed in young female mice treated with combined AngII and a high-fat diet that expression of *Arntl, Ciart, and Per2* was all modulated in the opposite direction we observed here (Aidara et al., 2024). The current knowledge of the control of cardiac clock genes does not provide much to explain this observation, and further studies are needed to both confirm and understand it better.

ARNTL and CLOCK are the central circadian clock transcription factors stimulating the expression of *Per* and *Cry* genes. In turn, PER and CRY protein products control ARNTL-CLOCK dimerization and activity. We observed that aging was associated with decreased expression of *Arntl* and the increase of *Ciart* (*Chrono*), *Per2*, and *Cry2* genes. *Clock* gene expression remained unchanged with age. A decrease of ARNTL protein (or a change of its rhythmicity) accompanied by increased expression (or shift in rhythmicity) of its repressors, such as CHRONO, PER2, or CRY2, could have effects on the cyclical controls of many genes in the heart. It may impair its adaptation to an insult.

ARNTL (BMAL1) has been linked to cardiac hypertrophy in previous publications. In rats with abdominal aorta coarctation, myocardial expression of *Arntl, Clock*, and *Rev-Erb α* (*Nr1d1*) genes were all reduced, whereas expression of repressors, such as *Per1/2* and *Cry1/2*, was increased (Giresi et al., 2005). Ablation of *Arntl* in mice was associated with a protective role in the development of cardiac hypertrophy (He et al., 2021; Yu, Ren & Dong, 2022; Liang et al., 2022; Herichová et al., 2013). Several mechanisms for this protective role have been proposed, including calcium management or autophagy.

## Study Limitations

We can identify several limitations to this study. Although we mentioned HFpEF murine models as a potential objective for this study, our animals needed to be fully characterized for the HFpEF phenotype. In older animals, we observed concentric hypertrophy, preserved EF, diastolic alterations, mostly left atrial dilation and hypertrophy, and several Doppler parameters. We did not submit the animals to exercise exhaustion protocols, and lung weight remained within the normal range. We did not incorporate metabolic stress such as a high-fat diet as in other recently developed HFpEF models (Withaar et al., 2021a; Schiattarella et al., 2019; Withaar et al., 2021b; Tong et al., 2019; Matsiukevich et al., 2023; Aidara et al., 2024).

Our transcriptomic studies were performed on total LV or LA RNA, so we must remain cautious about concluding that our observations can provide a cell-based explanation for the LV or LA function alterations.

Sham surgery was performed without the placement of a vehicle-filled osmotic minipump in all animals. Previous studies have shown that this had not caused differences in the heart (Matsiukevich et al., 2023; Aidara et al., 2024).

## Conclusion

In this study, we report a new mouse model of cardiac hypertensive disease for which the following variables were studied: old age, timing of gonadectomy, and AngII infusion at different stages of life. We hope this model may help better understand this frequent disease in aging women.

##  Supplemental Information

10.7717/peerj.17434/supp-1 Supplemental Information 1 Supplemental figures and tables

10.7717/peerj.17434/supp-2 Supplemental Information 2 Raw data to Figs. 1 to 5

10.7717/peerj.17434/supp-3 Supplemental Information 3 Processed data from left atrial RNA Seq for each sample

10.7717/peerj.17434/supp-4 Supplemental Information 4 Processed data for left ventricle RNA Seq for each sample

10.7717/peerj.17434/supp-5 Supplemental Information 5 Arrive 2.0 author checklist

## Additional Information and Declarations

### Data Availability

The following information was supplied regarding data availability: The data is available at NCBI GEO: GSE240171, GSE250052, and GSE250053.
