# Supplementary material for: A murine model of hypertensive heart disease in older women
Source: PeerJ. 2024 May 23;12:e17434. doi: 10.7717/peerj.17434 (PMC11128216; doi:10.7717/peerj.17434)
Supplement: Supplemental Information 1 [file peerj-12-17434-s001.pdf]

A murine model of hypertensive heart disease in elderly women.

Audrey Morin-Grandmont, Élisabeth Walsh-Wilkinson, Sara-Ève Thibodeau, Dominique Boudreau, Marie Arsenault, Yohan Bossé, Jacques Couet*
Groupe de recherche sur les valvulopathies, Centre de recherche de l'Institut universitaire de cardiologie et de pneumologie de Québec, Université Laval, Québec City, Québec, Canada

*: Correspondence to jacques.couet@med.ulaval.ca

Table S1. Echo data from young 4-month-old mice receiving a continuous AngII infusion for 14 or 28 days. Results are expressed as the mean ± SEM for the indicated number of animals. Statistical differences between CTRL and AngII (AngII) mice were analyzed using Student's T-test. a: $p<0.05$ and d: $p<0.0001$ between groups.

Parameters	Ctrl N=8	Ang 14d N=8	AngII 28d N=8
M-Mode			
EDD, mm	3,7 ± 0,04	3,6 ± 0,09	3.7 ± 0,12
ESD, mm	2,7 ± 0,07	2,3 ± 0,10	2,6 ± 0,12
PW, mm	0,70 ± 0,016	0,82 ± 0,030d	0,88 ± 0,023d
IVS, mm	0,66 ± 0,030	0,82 ± 0,026d	0,83 ± 0,023d
RWT	0,39 ± 0,015	0,46 ± 0,013c	0,47 ± 0,018d
Simpson's			
SV, mm	26.0 ± 1,42	27.6± 1,33	25,3 ± 2.15
EF, %	56,4 ± 2.15	59,6 ± 3,26	54,5 ± 2.19
HR, bpm	438 ± 19.8	454 ± 19.1	498 ± 9.48
CO, ml/min	11,8 ± 0,89	12.5 ± 0,72	13,2 ± 1.06
EDV, µl	46,1 ± 1,57	46,2 ± 1,87	46.2 ± 3.43
ESV, µl	20.1 ± 1,16	19,2 ± 2.51	25,3 ± 2.15
Doppler			
E, mm/s	572 ± 27,5	635 ± 27,9	597 ± 39.8
A, mm/s	356 ± 22,6	334 ± 19,8	367 ± 23,8
E/A	1,64 ± 0,093	1,92 ± 0,072	1,65 ± 0,112
E', mm/s	-26,2 ± 1,68	-27,2 ± 1,95	-26,5 ± 1,66
A', mm/s	-17,9 ± 0,95	-17,7 ± 1.08	-18,6 ± 1,49
E/E'	-22,1 ± 0,78	-23,8 ± 1.15	-22,7 ± 1.05
E'/A'	1,46 ± 0,051	1,54 ± 0,067	1,45 ± 0,095

Table S2: Echo data from adult mice treated or not with AngII. Left ventricle parameters were measured in 12-month-old female (F) mice, ovariectomized (Ovx) or not, and treated or not for 28 days with AngII. Results are expressed as the mean ± SEM for the indicated number of animals. Statistical differences between Non-Ovx and Ovx mice, and between CTRL and AngII mice were analyzed using two-way ANOVA and Holm-Sidak post-test for multiple comparison. a: $p<0.05$, b: $p<0.01$, c: $p<0.001$ and d: $p<0.0001$ between Ovx group and its corresponding Control group (F12 or F12 AngII, respectively). e: $p<0.05$, f: $p<0.01$, g: $p<0.001$ and h: $p<0.0001$ between an AngII group and its corresponding Control group (F12 or F12 Ovx, respectively).

Parameters	F12 N=12	F12 AngII N=8	F12 Ovx N=11-12	F12 Ovx AngII N=8
M-Mode				
EDD, mm	3,8 ± 0,07	3,4 ± 0,05f	3,9 ± 0,08	3,8 ± 0,07a
ESD, mm	2,6 ± 0,08	2,1 ± 0,07f	2,7 ± 0,10	2,7 ± 0,07c
PW, mm	0,84 ± 0,018	1,13 ± 0,026h	0,92 ± 0,022a	1,02 ± 0,044a,e
IVS, mm	0,76 ± 0,022	0,89 ± 0,020f	0,83 ± 0,023	0,91 ± 0,036
RWT	0,42 ± 0,011	0,59 ± 0,015h	0,45 ± 0,016	0,51 ± 0,027a,e
Simpson's				
SV, mm	35,8 ± 1,93	26,1 ± 0,84f	33,4 ± 1,78	26,6 ± 1,95e
EF, %	65,8 ± 1,64	67,1 ± 2,25	63,2 ± 1,55	58,5 ± 1,88a
HR, bpm	482 ± 6,1	510 ± 6,4	477 ± 7,0	520 ± 16,9e
CO, ml/min	17,2 ± 0,85	13,3 ± 0,56e	15,9 ± 0,77	13,8 ± 0,86
EDV, µl	54,8 ± 3,22	39,2 ± 2,05f	52,9 ± 2,45	45,4 ± 2,66
ESV, µl	19,0 ± 1,73	13,1 ± 1,57	19,5 ± 1,25	18,8 ± 1,23
Doppler				
E, mm/s	640 ± 10,0	627 ± 12,4	662 ± 22,1	611 ± 43,5
A, mm/s	414 ± 8,0	407 ± 16,1	433 ± 18,6	394 ± 26,2
E/A	1,55 ± 0,026	1,56 ± 0,057	1,54 ± 0,054	1,55 ± 0,045
E’, mm/s	-28,2 ± 0,73	-21,9 ± 1,25g	-26,9 ± 0,67	-27,7 ± 1,53b
A’, mm/s	-16,1 ± 0,59	-14,5 ± 0,98	-17,9 ± 1,00	-18,2 ± 1,48
E/E’	-22,8 ± 0,56	-29,3 ± 1,58g	-24,4 ± 1,04	-22,1 ± 1,12c
E’/A’	1,77 ± 0,074	1,52 ± 0,034e	1,54 ± 0,067	1,55 ± 0,069

Table S3: Echo data from elderly mice treated or not with AngII. Left ventricle parameters were measured in 24-month-old female (F) mice, ovariectomized (Ovx) or not, and treated or not for 28 days with AngII. Results are expressed as the mean ± SEM for the indicated number of animals. Statistical differences between Non-Ovx and Ovx mice, and between CTRL and AngII mice were analyzed using two-way ANOVA and Holm-Sidak post-test for multiple comparison. a: p<0.05 between Ovx group and its corresponding Control group (F24 or F24 AngII, respectively). e: p<0.05, f: p<0.01, and g: p<0.001 between an AngII group and its corresponding Control group (F24 or F24 Ovx, respectively).

Parameters	F24<br>N=7-8	F24 AngII<br>N=7	F24 Ovx<br>N=8	F24 Ovx AngII<br>N=7-8
M-Mode				
EDD, mm	4,0 ± 0,09	3,6 ± 0,09e	3,8 ± 0,04	3,6 ± 0,07
ESD, mm	2,6 ± 0,08	2,5 ± 0,11	2,6 ± 0,08	2,2 ± 0,08e
PW, mm	0,97 ± 0,045	0,98 ± 0,026	0,86 ± 0,022	1,08 ± 0,069f
IVS, mm	0,88 ± 0,030	0,94 ± 0,035	0,77 ± 0,016a	0,95 ± 0,034g
RWT	0,47 ± 0,021	0,53 ± 0,012	0,42 ± 0,012	0,56 ± 0,030g
Simpson's				
SV, mm	36,8 ± 2,31	30,0 ± 2,29	31,8 ± 1,09	27,4 ± 1,46
EF, %	59,4 ± 2,37	57,4 ± 1,91	56,1 ± 1,78	54,9 ± 2,84
HR, bpm	480 ± 30,0	521 ± 15,7	489 ± 6,2	524 ± 16,9
CO, ml/min	16,3 ± 2,15	15,6 ± 1,14	15,5 ± 0,46	14,2 ± 0,64
EDV, µl	61,9 ± 2,46	52,0 ± 2,90	56,9 ± 1,75	50,5 ± 3,34
ESV, µl	25,1 ± 1,56	22,0 ± 1,23	25,1 ± 1,55	23,2 ± 2,68
Doppler				
E, mm/s	614 ± 41,9	561 ± 27,5	626 ± 18,2	627 ± 53,4
A, mm/s	396 ± 26,1	370 ± 26,3	418 ± 10,0	431 ± 38,8
E/A	1,55 ± 0,046	1,53 ± 0,054	1,51 ± 0,059	1,46 ± 0,026
E’, mm/s	-29,6 ± 1,99	-25,3 ± 1,23	-28,2 ± 0,96	-25,6 ± 1,64
A’, mm/s	-19,5 ± 2,02	-17,3 ± 0,46	-18,1 ± 0,56	-16,6 ± 1,14
E/E’	-20,9 ± 1,40	-22,5 ± 1,58	-22,3 ± 0,50	-24,7 ± 1,91
E’/A’	1,56 ± 0,096	1,46 ± 0,044	1,56 ± 0,051	1,55 ± 0,036

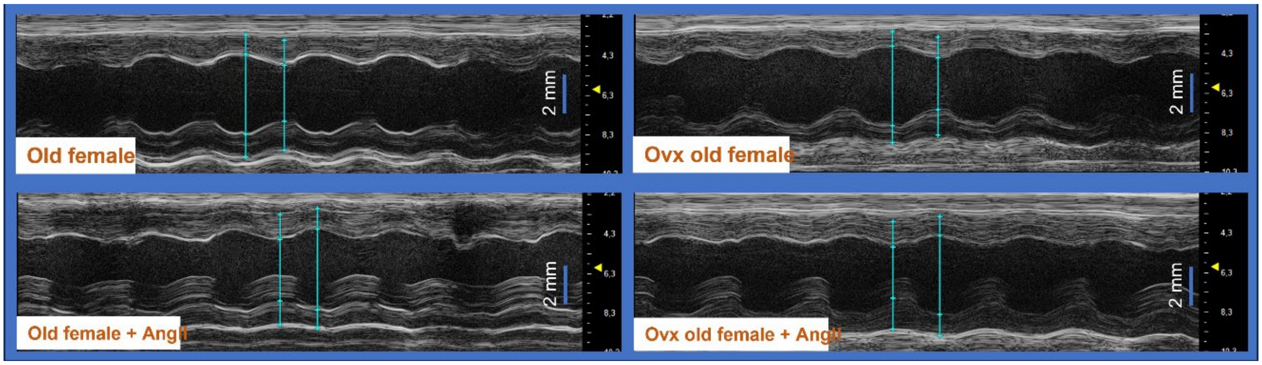

Figure S1. Representative M-mode echo images of the LV of old females (Ovx or not) treated or not with AngII. Light blue lines represent diastolic and systolic measurements of LV wall thickness and chamber diameter. Scaling bar: 2 mm. The images from young mice go from 2.2 to 9.3mm and those from old mice from 2.2 to 10.3 mm.

Table S4 Gene ontology (GO) list of biological processes from differentially expressed left ventricle or left atrial genes by AngII in old female mice (Ovx or not) compared to old mice. The list is limited to the top 10 biological processes which have the lowest false discovery rate. Number (Nb) of genes included for each category and fold enrichment over expected representation. In bold face, categories present in every comparison.

**GO biological process**	Nb of genes	Fold enrichment	False discovery rate
**LV Old mice vs. Old AngII**	**149**		
**extracellular structure organization**	22	11.57	5.74E-13
external encapsulating structure organization	22	11.57	8.61E-13
extracellular matrix organization	22	11.65	1.49E-12
anatomical structure morphogenesis	51	3.18	9.62E-11
collagen fibril organization	12	31.78	1.10E-10
tissue development	44	3.51	3.21E-10
animal organ development	54	2.68	8.50E-09
**developmental process**	80	2.01	1.21E-08
**anatomical structure development**	76	2.04	2.93E-08
regulation of multicellular organismal process	56	2.50	2.98E-08

**GO biological process**	Nb of genes	Fold enrichment	False discovery rate
**LV Old Ovx mice vs. Old Ovx AngII**	**187**		
regulation of multicellular organismal process	71	2.43	3.83E-09
animal organ development	60	2.28	2.54E-06
**anatomical structure development**	88	1.81	2.57E-06
**extracellular structure organization**	17	6.85	2.77E-06
**developmental process**	93	1.79	3.05E-06
external encapsulating structure organization	17	6.85	3.23E-06
extracellular matrix organization	17	6.90	3.49E-06
tissue development	45	2.75	4.14E-06
positive regulation of multicellular organismal process	44	2.62	5.80E-06
system development	65	2.08	5.88E-06

**GO biological process**	Nb of genes	Fold enrichment	False discovery rate
**LA Old mice vs. Old AngII**	**214**		
anatomical structure morphogenesis	63	2.73	1.63E-09
multicellular organism development	89	2.11	3.08E-09
system development	78	2.27	5.06E-09
**anatomical structure development**	102	1.90	7.97E-09
**developmental process**	105	1.83	2.14E-08
multicellular organismal process	122	1.62	4.04E-07
cell adhesion	31	3.50	5.12E-06
**extracellular structure organization**	17	6.22	7.59E-06
regulation of cell communication	69	2.00	8.19E-06
external encapsulating structure organization	17	6.22	8.35E-06

GO biological process	Nb of genes	Fold enrichment	False discovery rate
**LA Old Ovx mice vs. Old Ovx AngII**	**413**		
system development	139	2.06	2.88E-13
anatomical structure morphogenesis	105	2.32	3.77E-12
multicellular organism development	153	1.85	1.62E-11
**developmental process**	187	1.67	4.20E-11
**anatomical structure development**	177	1.69	1.25E-10
animal organ development	115	2.03	4.68E-10
tissue development	82	2.32	4.11E-09
**extracellular structure organization**	28	5.23	1.05E-08
extracellular matrix organization	28	5.27	1.12E-08
regulation of biological quality	114	1.91	1.14E-08

Table S5 Gene ontology (GO) list of cellular components from differentially expressed left ventricle or left atrial genes by AngII in old female mice (Ovx or not) compared to old mice. The list is limited to the top 10 biological processes which have the lowest false discovery rate. Number (Nb) of genes included for each category and fold enrichment over expected representation. In bold face, categories present in every comparison.

GO cellular component	Nb of genes	Fold enrichment	False discovery rate
**LV Old mice vs. Old AngII**	**149**		
**external encapsulating structure**	39	10.82	2.80E-25
**extracellular region**	73	4.09	3.00E-25
**extracellular matrix**	39	10.86	4.90E-25
**extracellular space**	62	4.61	3.58E-23
**collagen-containing extracellular matrix**	33	12.14	9.14E-23
**cell periphery**	84	2.00	5.70E-10
collagen trimer	11	19.78	9.69E-09
basement membrane	11	13.93	2.63E-07
banded collagen fibril	4	48.56	7.02E-04
fibrillar collagen trimer	4	48.56	7.80E-04

GO cellular component	Nb of genes	Fold enrichment	False discovery rate
**LV Old Ovx mice vs. Old Ovx AngII**	**187**		
**external encapsulating structure**	37	7.87	9.43E-19
**extracellular matrix**	37	7.90	1.67E-18
**collagen-containing extracellular matrix**	32	9.02	1.07E-17
**extracellular region**	71	3.05	1.05E-15
**extracellular space**	57	3.25	4.35E-13
**cell periphery**	99	1.80	1.47E-08
collagen trimer	9	12.41	3.06E-05
basement membrane	8	7.77	3.17E-03

**GO cellular component**	Nb of genes	Fold enrichment	False discovery rate
**LA Old mice vs. Old AngII**	**214**		
**external encapsulating structure**	35	6.76	1.77E-15
**extracellular matrix**	35	6.78	3.17E-15
**collagen-containing extracellular matrix**	29	7.42	1.29E-13
**extracellular region**	66	2.57	1.73E-10
**cell periphery**	111	1.84	3.43E-10
**extracellular space**	53	2.74	4.98E-09
collagen trimer	8	10.01	7.38E-04
supramolecular polymer	26	2.77	9.41E-04
supramolecular fiber	24	2.57	5.25E-03
basement membrane	8	7.05	5.67E-03

**GO cellular component**	Nb of genes	Fold enrichment	False discovery rate
**LA Old Ovx mice vs. Old Ovx AngII**	**413**		
**cell periphery**	222	1.87	3.76E-22
**external encapsulating structure**	57	5.62	9.50E-22
**extracellular matrix**	57	5.64	1.20E-21
**extracellular region**	129	2.57	4.22E-21
**extracellular space**	102	2.69	2.64E-17
**collagen-containing extracellular matrix**	44	5.75	5.87E-17
plasma membrane	172	1.58	1.07E-08
synapse	72	2.32	1.75E-08
plasma membrane region	63	2.36	1.44E-07
cell surface	54	2.55	1.91E-07

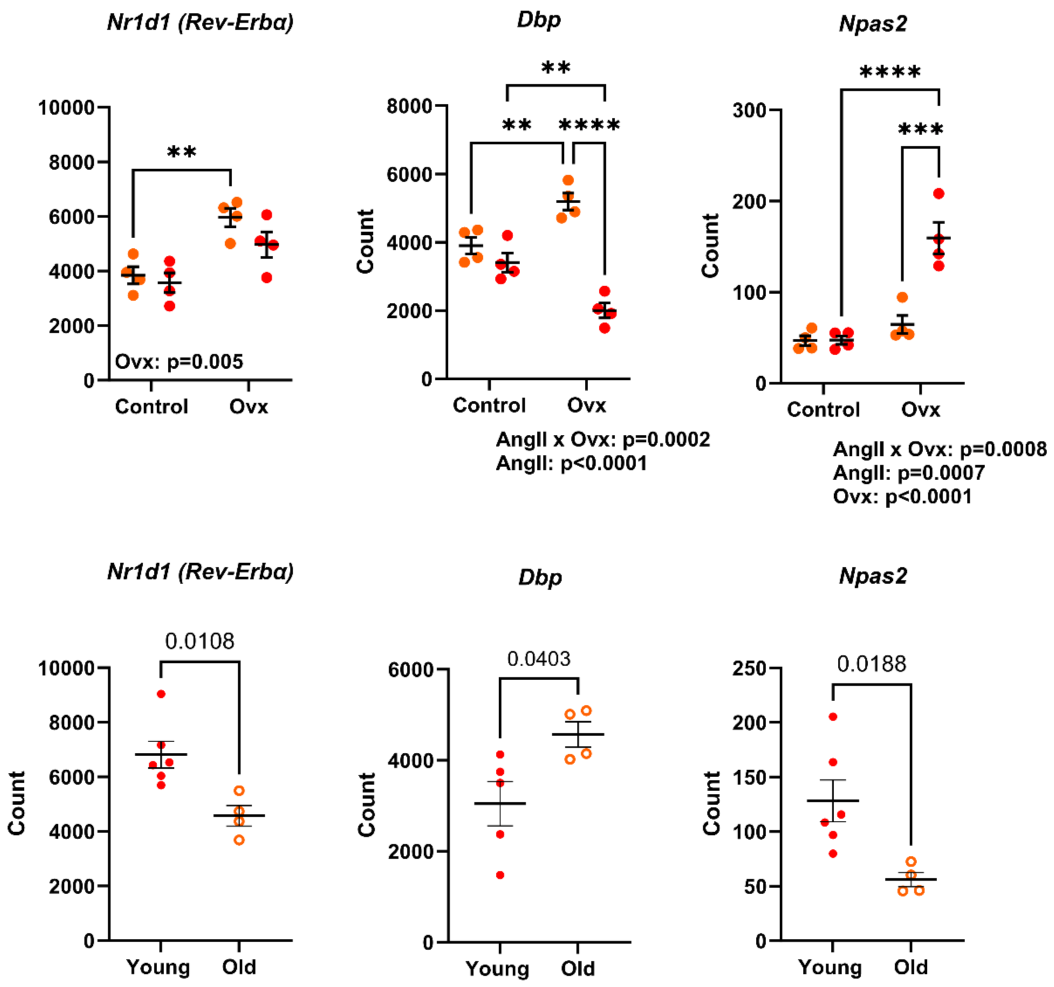

Figure S2. Expression of several circadian clock genes in young vs. old mice. Males (blue) and female (orange) mice. *Nr1d1 (Rev-Erbα)*: nuclear receptor subfamily 1 group D member 1, *Dbp*, D site albumin promoter binding protein and *Npas2*: neuronal PAS domain protein 2. Results are expressed as the mean ± SEM (n = 4). Statistical analysis by two-way ANOVA followed by Holm-Sidak post-test (up) or Student T-test (bottom). p values are indicated when under 0.05 below graphs. **: p<0.01, ***: p<0.001 and ****: p<0.0001 between indicated groups.

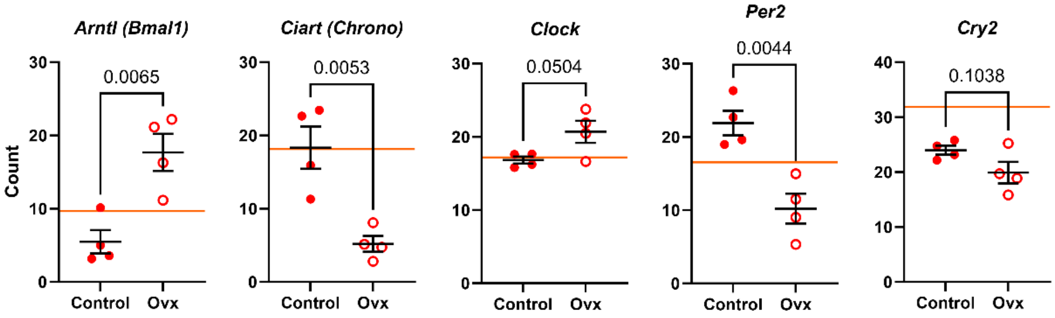

Figure S3. Expression of several circadian clock genes in the left atrium between elderly mice (control or Ovx). *Arntl*: Basic Helix-Loop-Helix ARNT Like 1, *Ciart*: Circadian Associated Repressor of Transcription, *Clock*: Clock Circadian Regulator, *Per2*: Period Circadian Regulator 2 and *Cry2*: Cryptochrome Circadian Regulator 2. The line represents the average expression of a given gene in young females.. Results are expressed as the mean ± SEM (n = 4-6). Statistical analysis by two-way ANOVA followed by Holm-Sidak post-test. Age and Sex p values are indicated when under 0.05 besides graphs. **: p<0.01, ***: p<0.001 and ****: p<0.0001 between indicated groups.
